# Supplementary material for: Splice-Junction-Based Mapping of Alternative Isoforms in the Human Proteome
Source: Cell Rep. Author manuscript; Available in PMC 2020 Jan 15. (PMC6961840; doi:10.1016/j.celrep.2019.11.026)
Supplement: 3 [file NIHMS1546469-supplement-3.zip › DF2/PXD0010154/Lung-112-Q9UPN9-FNEADSEVAQAGK.pdf]

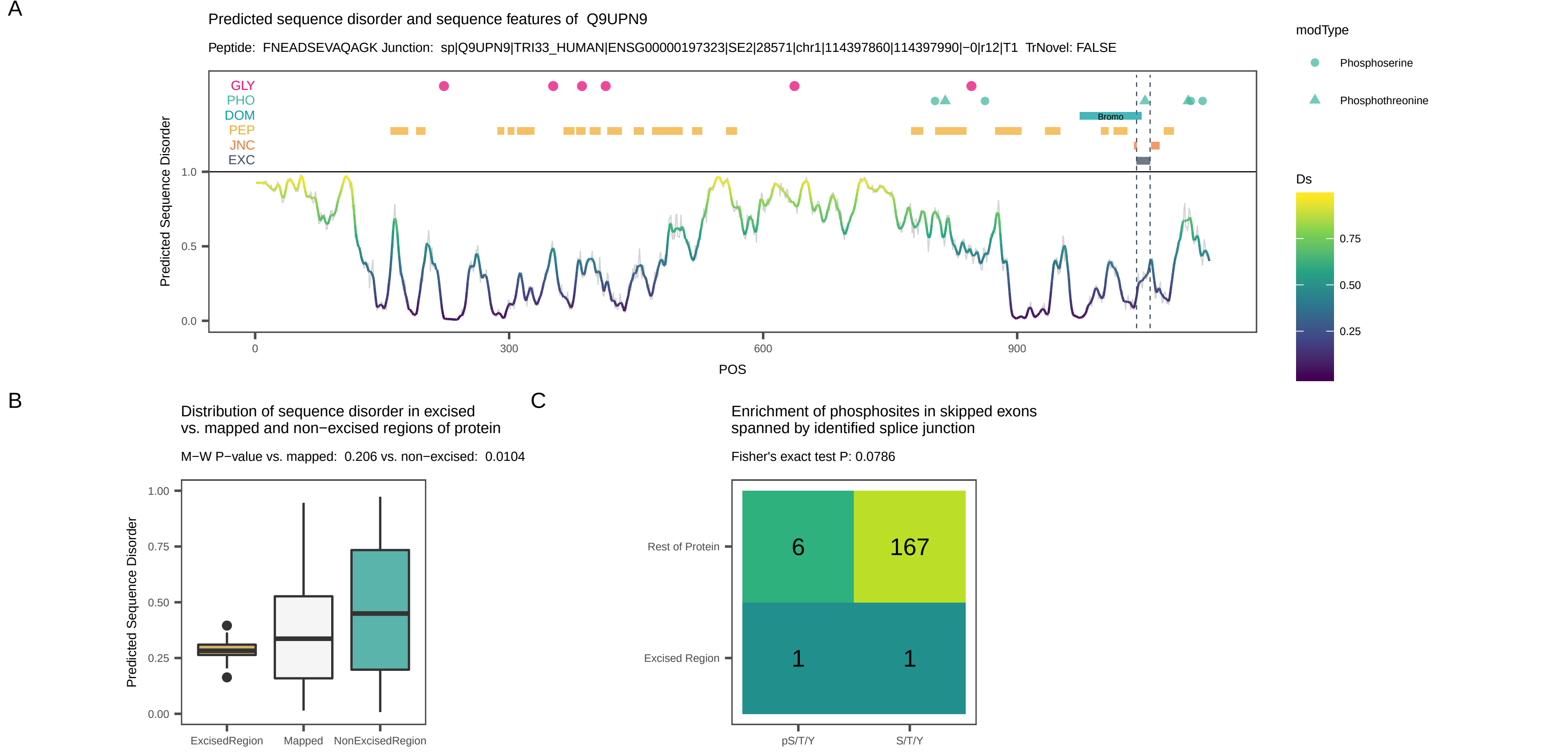
A
Predicted sequence disorder and sequence features of Q9UPN9
Peptide: FNEADSEVAQAGK Junction: sp|Q9UPN9|TRI33_HUMAN|ENSG00000197323|SE2|28571|chr1|114397860|114397990|−0|r12|T1 TrNovel: FALSE
GLY
PHO
DOM
PEP
JNC
EXC
Bromo
Predicted Sequence Disorder
1.0
0.5
0.0
0
300
600
900
POS
modType
Phosphoserine
Phosphothreonine
Ds
0.75
0.50
0.25
B
Distribution of sequence disorder in excised
vs. mapped and non−excised regions of protein
M−W P−value vs. mapped: 0.206 vs. non−excised: 0.0104
Predicted Sequence Disorder
1.00
0.75
0.50
0.25
0.00
ExcisedRegion
Mapped
NonExcisedRegion
C
Enrichment of phosphosites in skipped exons
spanned by identified splice junction
Fisher's exact test P: 0.0786
Rest of Protein
Excised Region
6
167
1
1
pS/T/Y
S/T/Y
